# High Velocity, Low‐Voltage Collective In‐Plane Switching in (100) BaTiO_3_ Thin Films

**DOI:** 10.1002/advs.202201530

**Published:** 2022-08-28

**Authors:** Trygve M. Ræder, Shuyu Qin, Michael J. Zachman, Rama K. Vasudevan, Tor Grande, Joshua C. Agar

**Affiliations:** ^1^ Department of Physics DTU Danmarks Tekniske Universitet Kgs. Lyngby 2800 Denmark; ^2^ Department of Materials Science and Engineering NTNU Norwegian University of Science and Technology Trondheim NO‐7491 Norway; ^3^ Department of Computer Science and Engineering Lehigh University Bethlehem PA 18015 USA; ^4^ The Center for Nanophase Materials Sciences Oak Ridge National Laboratory Oak Ridge TN 37831 USA; ^5^ Department of Materials Science and Engineering Lehigh University Bethlehem PA 18015 USA; ^6^ Department of Mechanical Engineering and Mechanics Drexel University Philadelphia PA 19104 USA

**Keywords:** barium titanate, capacitive hysteresis, ferroelectric switching, ferroelectrics, neural network, piezoresponse force microscopy

## Abstract

Ferroelectrics are being increasingly called upon for electronic devices in extreme environments. Device performance and energy efficiency is highly correlated to clock frequency, operational voltage, and resistive loss. To increase performance it is common to engineer ferroelectric domain structure with highly‐correlated electrical and elastic coupling that elicit fast and efficient collective switching. Designing domain structures with advantageous properties is difficult because the mechanisms involved in collective switching are poorly understood and difficult to investigate. Collective switching is a hierarchical process where the nano‐ and mesoscale responses control the macroscopic properties. Using chemical solution synthesis, epitaxially nearly‐relaxed (100) BaTiO_3_ films are synthesized. Thermal strain induces a strongly‐correlated domain structure with alternating domains of polarization along the [010] and [001] in‐plane axes and 90° domain walls along the [011] or [01$\bar{1}$] directions. Simultaneous capacitance–voltage measurements and band‐excitation piezoresponse force microscopy revealed strong collective switching behavior. Using a deep convolutional autoencoder, hierarchical switching is automatically tracked and the switching pathway is identified. The collective switching velocities are calculated to be ≈500 cm s^−1^ at 5 V (7 kV cm^−1^), orders‐of‐magnitude faster than expected. These combinations of properties are promising for high‐speed tunable dielectrics and low‐voltage ferroelectric memories and logic.

## Graphical abstract

Here, a technique for simultaneous capacitance–voltage measurements and band‐excitation piezoresponse force microscopy is introduced. In epitaxially nearly‐relaxed (100) BaTiO_3_ films, strongly‐correlated domain structure are observed using a deep convolutional autoencoder and hierarchical switching is automatically tracked. Fast collective switching velocities (500 cm s^−1^ at 7kV cm^−1^), significantly faster than expected in a strongly clamped film, are observed.

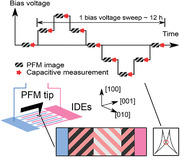

## 1 Introduction

The emergence of correlated domain dynamics in ferroelectrics have enabled low‐loss tunable dielectrics, negative capacitance, and high clock frequency.^[^ ^1^, ^2^, ^3^, ^4^, ^5^, ^6^ ^]^ The increasing need for memory and logic devices for extreme environments has made these properties important figures of merit for ferroelectric devices. Domain structure engineering is a powerful tool to tune these properties, but there are challenges in correlating properties to collective switching dynamics.^[^ ^7^ ^]^

To understand collective switching dynamics a multitude of macroscopic and nanoscale characterization techniques have been employed. On the macroscale, ferroelectric hysteresis, positive‐up negative‐down (PUND),^[^ ^8^ ^]^ and capacitance–voltage measurements provide insight into collective switching dynamics. On the nanoscale, a range of scanning‐probe‐based switching spectroscopes have observed local switching dynamics.^[^ ^9^, ^10^, ^11^ ^]^ For example, studies have utilized a conductive cantilever‐mounted tip to apply a bipolar triangular switching waveform while imaging the domain structure using piezoresponse force microscopy (PFM).^[^ ^12^, ^13^, ^14^ ^]^ When measuring switching dynamics directly under the tip, it is not possible to observe collective switching behavior as switching occurs over length scales of micrometers.^[^ ^15^ ^]^ Furthermore, these measurements are not applicable to in‐plane switching dynamics as it is difficult to determine and decouple lateral electric fields when bias is applied between a conductive‐PFM tip and bottom electrode. From a device perspective, in‐plane polarization is favorable as this keeps the polarization in the plane where the elastic boundary conditions are enforced by the substrate, yielding stronger collective behavior. Tensile strain can produce different dense in‐plane domain structures,^[^ ^16^, ^17^ ^]^ while compressive strain produces monodomain states or domain structures with both in‐plane and out‐of‐plane components.^[^ ^18^ ^]^

One modern approach to investigate in‐plane switching is to use coplanar electrodes, as demonstrated in BiFeO_3_ thin films^[^ ^12^ ^]^ and LiNbO_3_^[^ ^19^ ^]^ single crystals. These studies investigate switching in a step‐wise fashion by alternating between voltage pulses and PFM scans. This approach cannot avoid domain structure relaxation between images and thus cannot reveal transient states stable under an applied voltage. In contrast, imaging the domain structure in situ, while a voltage is applied, better mimics operational conditions, but has so far only been performed using TEM.^[^ ^20^, ^21^ ^]^ TEM is plagued by the requirement that the sample is thin enough to be electron transparent, which relaxes the elastic boundary conditions along one axis in the film and introduces additional surfaces. It is vital for comparison that the film is under the same boundary conditions in microscopic and macroscopic characterization. Similar false conclusions may be drawn if different techniques are not performed at the same frequency, because important parameters such as the coercive field are frequency dependent. Finally, many ferroelectrics experience wake‐up and ageing behaviors,^[^ ^22^, ^23^ ^]^ which can lead to different results if experiments are not carried out concurrently. Combining data from different sources therefore requires that nondestructive experiments are conducted in situ.

Here, we develop an in situ correlated capacitance–voltage and band‐excitation PFM imaging technique to directly correlate collective switching dynamics to functional properties in a BaTiO_3_ thin film. Nearly‐relaxed epitaxially film are fabricated using chemical solution deposition producing a strongly coupled *a*_1_/*a*_2_ domain structure^[^ ^24^ ^]^ as the result of thermally‐induced tensile strain. Ferroelectric hysteresis loops reveals fast switching events, significantly faster than would be expected for clamped films.^[^ ^25^, ^26^, ^27^ ^]^ Simultaneous capacitance–voltage and band‐excitation piezoresponse force microscopy (PFM) measurements reveal strong collective switching behavior. Using a deep convolutional autoencoder we automatically track hierarchical switching and identify the switching pathway. We calculate the collective switching velocities to be ≈500 cm s^−1^ at 5 V (7 kV cm^−1^), orders‐of‐magnitude faster than expected. These combinations of properties are promising for high‐speed tunable dielectrics and high‐speed low‐voltage ferroelectric memories and logic.

## 2 Results

**Figure 1:**
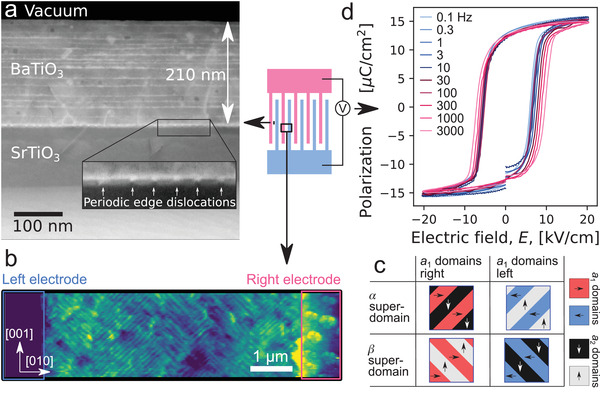
a) Annular dark field (ADF) STEM image of the 210  nm BaTiO_3_ film; Periodic edge dislocations at the interface are highlighted in the inset. b) Conventional PFM image (driving voltage applied to the tip) that shows the amplitude of the piezoresponse along the [001] direction. *a*_2_ domains appear as diagonal bright lines separated by dark *a*_1_ domains. The image shows the amplitude of *a*_2_. c) Types of superdomains in an *a*_1_/*a*_2_ domain structure. The superdomains are categorized based on whether the *a*_1_ domains have polarization left or right and the direction of the domain walls, with α superdomains having domain walls along the [011] direction, and β superdomains having domain walls along the [01−1] direction. d) Polarization‐electric field hysteresis loops from 0.1 Hz to 3 kHz.

BaTiO_3_ films were grown using chemical solution deposition. Each of the 16 layers of BaTiO_3_ were individually calcined to form an epitaxial film. Scanning transmission electron microscopy (STEM) images of the interface show individual layers (**Figure** **1**a). The epitaxial strain at the substrate–film interface is relaxed by edge dislocations. Further details are provided in ref. [28].

To understand the domain structure we conducted detailed PFM studies. PFM studies reveal an *a*_1_/*a*_2_ superdomains (Figure 1b,c) with alternating domains of polarization along the [010] and [001] in‐plane axes and 90° domain walls along the [011] or [01$\bar{1}$] directions.^[^ ^16^ ^]^ In‐plane ferroelectric hysteresis loops measured using interdigitated electrodes (IDEs) reveal square hysteresis loops with sharp switching at frequencies from 0.1 Hz to 3 kHz (Figure 1d).

We have estimated the collective switching velocity from the switching current and electrode spacing according to Equation (1).

(1) $$v={\left(\frac{1}{dP/dE_{\text{lf}}}-\frac{1}{dP/dE_{\text{hf}}}\right)}^{-1}{\frac{dE}{dt}}_{\text{hf}}\frac{a}{2P_{m}}$$

Here d*P*/d*E*_hf_ and d*P*/d*E*_lf_ are the maximum slopes of the high‐frequency and low‐frequency curves (Figure 1d), *a* is the electrode spacing and *P*_m_ is the maximum polarization. The difference in slope between high and low frequency measurements corrects for the small (<±0.2 µ m) nonuniformity in the electrode spacing across the IDE area (1×1 mm) and other similar features which cause $\frac{dP}{dE}$ to plateau at low frequencies. With this data (Figure 1d, Equation (1)) we estimate a domain wall velocity of ≈500 cm s^−1^. This is higher than velocities observed at the coercive field in bulk single‐crystals of BaTiO_3_,^[^ ^29^ ^]^ which is surprising due to the strong clamping that suppresses irreversible domain wall motion in epitaxial thin films,^[^ ^25^, ^26^, ^27^ ^]^ and we attribute this to the strongly correlated domain structure.

**Figure 2:**
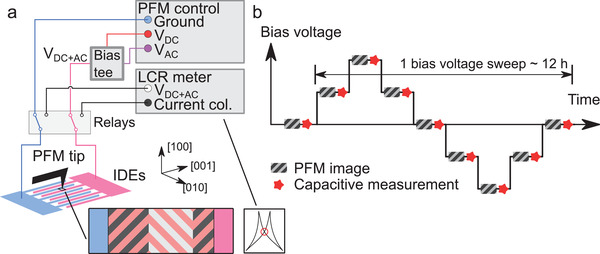
a) Illustration of the experimental setup. The driving potential, *V*_AC_, and bias, *V*_DC_, used to control the PFM were combined using a bias tee, and connected to the interdigitated electrodes via relays. By switching the relays, the IDEs would instead be connected to an LCR meter. b) Measurement scheme. The bias voltage was swept in a stepwise fashion, an at each step a BEPFM frame was recorded, followed by a capacitive measurement. A simplified schema with two steps on the rising edge and a single bias sweep is shown. For the experiment, 16 steps were used on the rising edge, and two bias sweeps were performed.

In situ images were collected by band‐excitation piezoresponse force microscopy (BEPFM)^[^ ^30^ ^]^ using the same IDEs. A DC bias voltage *V*_DC_ was applied together with the driving voltage *V*_AC_ to the electrodes (**Figure** **2**). The electric field distribution was confirmed using Kelvin‐probe force microscopy (KPFM—Section S1, Supporting Information).

Images were constructed by measuring the lateral deflection of the cantilever associated with the lateral piezoresponse driven by the band excitation waveform. The raw data in the time domain was translated to the frequency domain using a Fourier transformation. The frequency‐dependent piezoresponse was fit to an simple harmonic oscillator using a neural network approximate that was refined by least‐squares curve fitting.^[^ ^31^ ^]^

**Figure 3:**
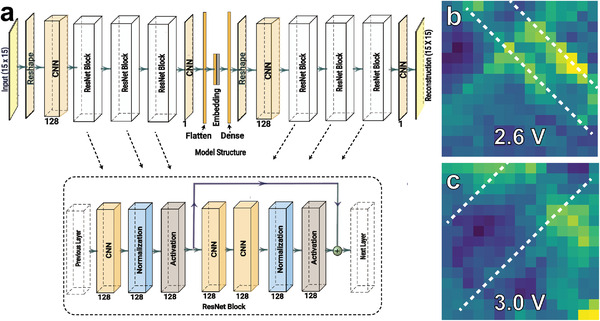
a) Schematic depiction of deep neural network architecture used to detect ferroelectric switching. b,c) Median image of primary switching event between 2.6 and 3.0 V.

To automate the detection of ferroelectric switching dynamics with minimal user bias we deployed a custom convolutional neural network (CNNs) concept (**Figure** **3**). Prior to training, each image was normalized to have a mean of zero (0) and a standard deviation of one (1). This eliminates image variations that occur due to reconstruction of the PFM tip during imaging. The data was parsed into 15×15 pixel images by convolving a kernel with a stride of one (1). These kernels were analyzed using a deep convolutional autoencoder. Autoencoders consist of three parts, an encoder, embedding layer, and decoder. In this architecture the encoder seeks to identify a compressed statistical distribution of the training data. This abstract representation is passed to the embedding layer which compresses this information into an interpretable latent space. The output of the embedding layer is then decoded by the decoder which seek to reconstruct the original image. Autoencoders are optimized to learn and identify function *f*(*x*) = *x*, with consideration for other regularization constraints.

In our model, the encoder ingests a single channel image using a convolutional layer with a kernel of three (3) and a stride of one (1). The output is then passed to three identical blocks. Each convolutional block has two parts. The first is a convolutional layer with a layer normalization and rectified linear activation functions (ReLu) as nonlinearities. The second part is a residual block that has two (2) convolutional layers, a layer normalization layer, and a ReLu activation function. The input of the residual block is added to the output of this block forming a residual network (ResNet). ResNets are commonly used to improve optimization and increase model performance.^[^ ^32^ ^]^ At the end of the encoder there is a single convolutional neuron which restores the dimensionality to a single (15 × 15) image. This downsampled image is then flattened and passed to a single fully‐connected layer that compressed each image to a vector of size 64.

The decoder is constructed as the inverse of the encoder. There is a fully‐connected layer that restores the embedding layer to a vector of size 225, which is then reshaped to a 15 × 15 image. This is followed by a convolutional layer, and three convolutional blocks with residual layers. The last layer of the model is a single convolutional neuron that restores the dimensionality to the original image size. The model was optimized using ADAM for 86 epochs reaching a final reconstruction loss of 0.06.

**Figure 4:**
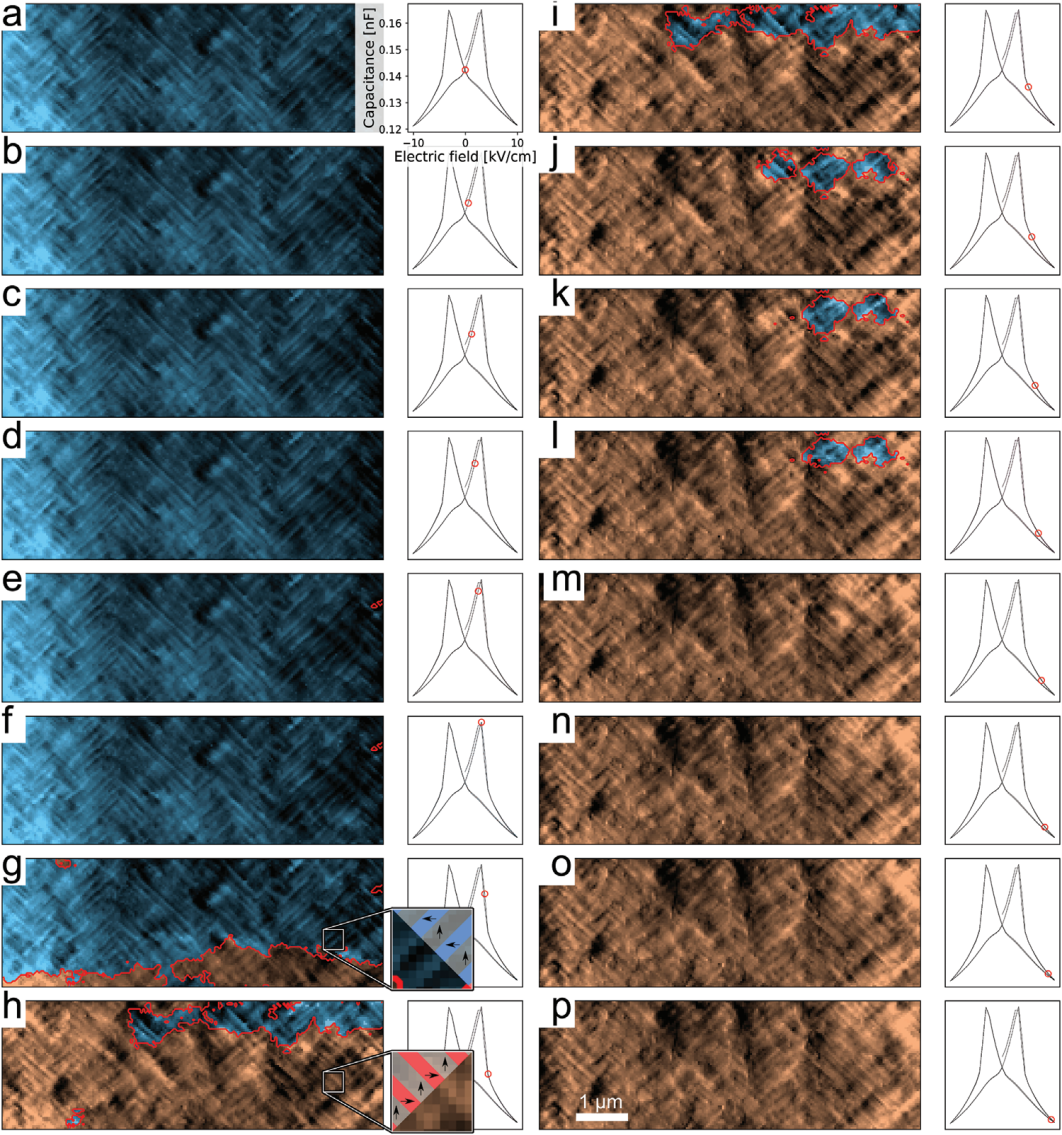
BEPFM images at different bias voltage. The corresponding capacitance is circled in the *C*–*V* curve associated with each PFM image. In the images, the color represents the polarization direction and the shade is the real part of the complex piezoresonse.

Following training the output from the embedding layer was extracted. This represents a compact representation of the ensemble of images. To determine the point of ferroelectric switching we identified the voltage step within each convolution where there was the largest change in the embedding. The time step where the embedding has the largest Euclidean distance between time steps in the latent space represents where the image, and thus the domain structure changed the most, a marker for ferroelectric switching. This was used to create the switching projections (**Figure** **4**). To confirm that the maximum change in the embedding represents a switching event, we, for each voltage step selected the median pixels from the embedding that were classified as switching. As an example, we show the change, from the median switching event as determined from the embeddings, in the primary switching event that occurred between voltages of 2.6 and 3 V (Figure 3a,b).

In the experiment, the voltage was cycled twice, switching the film four times (two times left to right, and two times right to left). All four switching events show similar dynamics, and all PFM images are included in Section S2, Supporting Information. PFM images recorded at increasing bias voltage during the third switching event are shown in Figure 4. From the neural network, we plot the polarization superimposed on the PFM image of the domain structure. Regions with the polarization aligned opposed to the electric field are shaded in blue (unswitched) while regions aligned with the electric field are shaded in red (switched). We indicate the capacitance using a red circle in the plot to the right of the domain structure image. No bias was applied in the image shown in Figure 4a. In Figure 4b–d sub‐coercive fields were applied and the capacitance increases significantly although no switching is observed. The domain structure in Figure 4e,f remained very similar to that in Figure 4a, but a nucleated domain of reverse polarization by the right electrode has been identified. Increasing the field past the coercive field, Figure 4g shows a significant switching event spanning between the electrodes in the bottom part of the frame. This is associated with a significant drop in the capacitance.

From Figure 4g,h major sections of the film switch and the capacitance drops further. At the left side of the image the film is now completely switched. However, the switched region splits and connects to the other electrode in the bottom right of the image and top‐right just out of the frame, leaving a smaller region of the film not‐yet‐switched. This major switching event is used to define the coercive field, 3.75±0.62 kV cm^−1^, where the uncertainty is given by the voltage step. By comparing the local domain structure in inset (g) and (h), it can be seen that switching rotates the domain walls by 90° from α superdomain to a β superdomain (described in Figure 1d) and identified by the neural network (Figure 3b,c). Switching continues from Figure 4h through m as the not‐yet‐switched region shrinks and eventually disappears. This demonstrates that switching continues beyond the coercive field, and nonswitched regions are present up to Figure 4l (1.5 times the coercive field). No significant changes to the domain structure is observed in Figure 4m–p.

## 3 Discussion

**Figure 5:**
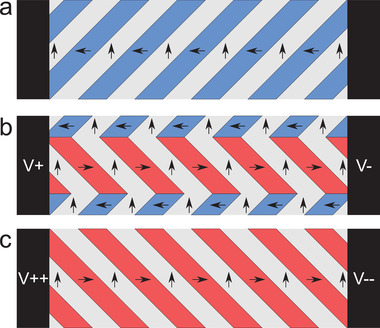
Sketch of the proposed switching mechanism where switching occurs as bands connecting the two electrodes. a) The poled state showing a single superdomain. b) By applying a potential difference to the electrodes, a band connecting the two electrodes is switched. When switching from an α superdomain to a β superdomain, no charged domain walls are introduced as the band switches. c) By applying a larger potential difference, the remainder of the film is switched.

These results demonstrate that nucleation takes place at the electrodes prior to reaching the coercive field. This is expected in a coplanar electrode geometry as the electric field will be highly concentrated at the edge of the electrodes.^[^ ^33^ ^]^ Domain switching in major parts of the film were only initiated after the maximum capacitance was reached, indicating that the coercive field did not align with a maximum in the average dielectric constant. This is expected as the largest dielectric response should be at the precipice of ferroelectric switching. The drop in the capacitance as the film switches corresponds roughly to the fraction of the film where domain switching had occurred. This suggests that the capacitance is determined by the volume fraction of domains pointing right and left, as well as the applied bias. Domains pointing opposite of the applied bias increases the capacitance, while domains pointing along the applied bias decreases the capacitance. We observe from our studies that the superdomain structure switches as a nearly‐coherent unit. This is consistent with previous reports observed in multiple systems.^[^ ^34^ ^]^ The Landau–Lifshitz–Kittel domain model^[^ ^34^, ^35^, ^36^ ^]^ can therefore not be used to describe the switching. A preferable switching mechanism is observed, where multiple superdomains switch together as a band connecting the two electrodes. Based on our macroscopic studies (Figure 1) these domain appear to switch in a single collective event. Switching in a band connecting the two electrodes is shown in **Figure** **5**. In the sketch it can be seen that when a band switches by a 90° rotation of the domain walls (switching from an α to a β superdomain), the band may switch without introducing charged domain walls.

Domain pinning was observed in the top right of the image (Figure 4, where switching was observed at much greater fields than in the remainder of the film. The area was surrounded by regions that had already switched, and could therefore not switch as a band connecting the two electrodes. This precludes collective switching mechanisms, effectively pinning the domains. Switching induces a 90° rotation of the domain walls in the superdomains, and when large sections of the film has been switched, it can be seen that the distribution of superdomains is similar before and after switching. Whether the distribution of superdomains is preserved because it is favored by an immobile defect, or because the kinetics of the switching event preserves the superdomains remains an open question. That being said, switching does not preserve the superdomains perfectly (Section S3, Supporting Information).

## 4 Conclusion

A novel PFM configuration for studying in‐plane switching dynamics of thin films is developed. Fast switching dynamics were established based on *P*–*V* curves. Correlated *C*–*V* curves and PFM images of a BaTiO_3_ thin film with IDEs were presented. Neural networks were used in both preprocessing of the data and to infer the polarization direction and switching dynamics. Switching occurred in bands that connected the two electrodes, and was associated with 90° rotation of domain walls inside superdomains, while the distribution of superdomains was largely preserved. Simultaneous capacitance–voltage measurements indicated that the capacitance peaked before the coercive field was reached. The shape of the *C*–*V* curves was a result of the switching kinetics, where the capacitance decreased as sections of the film switched. We determine that the collective switching mechanism drives low‐voltage 5 V (7 kV cm^−1^), fast‐switching at velocities greater than ≈500 cm s^−1^, orders‐of‐magnitude faster than expected. These combinations of properties are promising for high‐speed tunable dielectrics and high‐speed low‐voltage ferroelectric memories and logic.

## 5 Experimental Section

### Experimental

BaTiO_3_ films were synthesized on (001)‐oriented SrTiO_3_ using aqueous chemical solutions from titanium‐isopropoxide and barium nitrate precursors.^[^ ^24^ ^]^ Epitaxy was induced by heating each layer to 1000 °C. Reciprocal space mapping has shown residual tensile strain associated with a relaxed film under thermal strain.^[^ ^16^ ^]^ 16 layers produced 210 nm of BaTiO_3_. The strain relaxation was attributed to edge dislocations induced at high temperature that were observed using cross‐sectional transmission electron microscopy.

PFM images were measured using BEPFM with the driving voltage connected to the electrodes. The PFM tip was electronically insulated from the circuit so that it would not short‐circuit the in situ voltage or otherwise disturb the domain structure. A multifunction PXI system (National Instruments) running a custom Labview program was used to generate the BE waveform (*V*_AC_), while the DC bias (*V*_DC_) was supplied from the PFM control unit via a 10× amplifier (F10A, FLC Electronics AB, Göteborg, Sweden). The experimental configuration is illustrated in Figure 2a. The relays were controlled by a multifunctional PXI system. An Agilent E4980A LCR meter (Agilent Technologies, Santa Clara, CA, USA) was used for capacitive measurements (1 kHz). An ElectriMulti75‐G (BudgetSensors, Sofia, Bulgaria) PFM tip was used. A Python script was used to automate the image acquisition, capacitive measurements, and adjusting the bias voltage according to the measurement scheme illustrated in Figure 2b.

### Neural Network

Neural networks were trained in Pytorch as described in the main text. Models were trained on a Lambda Labs workstation with 128 GB ram and two (2) Nvidia Titan RTX graphics processing units. Models were trained with a batch size of 512. The models were optimized using ADAM with a learning rate of 3 × 10^−5^.

## Conflict of Interest

The authors declare no conflict of interest.

## Supporting information

Supporting Information

## Data Availability

The data that support the findings of this study are openly available in Zenodo at https://doi.org/10.5281/zenodo.5529742, reference number 5529742.

## References

[1] Z. Gu , S. Pandya , A. Samanta , S. Liu , G. Xiao , C. J. Meyers , A. R. Damodaran , H. Barak , A. Dasgupta , S. Saremi , A. Polemi , L. Wu , A. A. Podpirka , A. Will‐Cole , C. J. Hawley , P. K. Davies , R. A. York , I. Grinberg , L. W. Martin , J. E. Spanier , Nature 2018, 560, 622. 30127406 10.1038/s41586-018-0434-2

[2] P. Zubko , J. C. Wojdeł , M. Hadjimichael , S. Fernandez‐Pena , A. Sené , I. Luk'yanchuk , J.‐M. Triscone , J. Íñiguez , Nature 2016, 534, 524. 27296225 10.1038/nature17659

[3] A. I. Khan , K. Chatterjee , J. P. Duarte , Z. Lu , A. Sachid , S. Khandelwal , R. Ramesh , C. Hu , S. Salahuddin , IEEE Electron Device Lett. 2016, 37, 111.

[4] W. Gao , A. Khan , X. Marti , C. Nelson , C. Serrao , J. Ravichandran , R. Ramesh , S. Salahuddin , Nano Lett. 2014, 14, 5814. 25244689 10.1021/nl502691u

[5] R. Xu , S. Liu , S. Saremi , R. Gao , J. J. Wang , Z. Hong , H. Lu , A. Ghosh , S. Pandya , E. Bonturim , Z. H. Chen , L. Q. Chen , A. M. Rappe , L. W. Martin , Nat. Commun. 2019, 10, 1282. 30894533 10.1038/s41467-019-09207-9 PMC6427024

[6] S. Manipatruni , D. E. Nikonov , C.‐C. Lin , T. A. Gosavi , H. Liu , B. Prasad , Y.‐L. Huang , E. Bonturim , R. Ramesh , I. A. Young , Nature 2019, 565, 35. 30510160 10.1038/s41586-018-0770-2

[7] S. Das , Z. Hong , M. McCarter , P. Shafer , Y.‐T. Shao , D. A. Muller , L. W. Martin , R. Ramesh , APL Mater. 2020, 8, 120902.

[8] S. Divilov , H.‐C. Hsing , M. H. Yusuf , A. Gura , J. A. Garlow , M.‐G. Han , M. Stengel , J. Bonini , P. Chandra , K. M. Rabe , M. Fernandez Serra , M. Dawber , *arXiv:2011.06082* 2020.

[9] J. C. Agar , B. Naul , S. Pandya , S. van der Walt , J. Maher , Y. Ren , L.‐Q. Chen , S. V. Kalinin , R. K. Vasudevan , Y. Cao , J. S. Bloom , L. W. Martin , Nat. Commun. 2019, 10, 4809. 31641122 10.1038/s41467-019-12750-0 PMC6805893

[10] A. R. Damodaran , S. Pandya , J. C. Agar , Y. Cao , R. K. Vasudevan , R. Xu , S. Saremi , Q. Li , J. Kim , M. R. McCarter , L. R. Dedon , T. Angsten , N. Balke , S. Jesse , M. Asta , S. V. Kalinin , L. W. Martin , Adv. Mater. 2017, 29, 37. 10.1002/adma.201702069 28758269

[11] J. C. Agar , A. R. Damodaran , M. B. Okatan , J. Kacher , C. Gammer , R. K. Vasudevan , S. Pandya , L. R. Dedon , R. V. K. Mangalam , G. A. Velarde , S. Jesse , N. Balke , A. M. Minor , S. V. Kalinin , L. W. Martin , Nat. Mater. 2016, 15, 549. 26878312 10.1038/nmat4567

[12] B. N. Balke , M. Gajek , A. K. Tagantsev , L. W. Martin , Y.‐h. Chu , R. Ramesh , S. V. Kalinin , Adv. Funct. Mater. 2010, 20, 3466.

[13] N. A. Polomoff , R. N. Premnath , J. L. Bosse , B. D. Huey , J. Mater. Sci. 2009, 44, 5189.

[14] H. J. Lee , T. Shimizu , H. Funakubo , Y. Imai , O. Sakata , S. H. Hwang , T. Y. Kim , C. Yoon , C. Dai , L. Q. Chen , S. Y. Lee , J. Y. Jo , Phys. Rev. Lett. 2019, 123, 217601. 31809179 10.1103/PhysRevLett.123.217601

[15] X. Lu , Z. Chen , Y. Cao , Y. Tang , R. Xu , S. Saremi , Z. Zhang , L. You , Y. Dong , S. Das , H. Zhang , L. Zheng , H. Wu , W. Lv , G. Xie , X. Liu , J. Li , L. Chen , L.‐Q. Chen , W. Cao , L. W. Martin , Nat. Commun. 2019, 10, 3951. 31477695 10.1038/s41467-019-11825-2 PMC6718682

[16] T. M. Raeder , T. S. Holstad , I.‐E. Nylund , M.‐A. Einarsrud , J. Glaum , D. Meier , T. Grande , AIP Adv. 2021, 11, 025016.

[17] A. R. Damodaran , J. C. Agar , S. Pandya , Z. Chen , L. Dedon , R. Xu , B. Apgar , S. Saremi , L. W. Martin , J. Phys. Condens. Matter 2016, 28, 263001. 27187744 10.1088/0953-8984/28/26/263001

[18] R. Waser , N. A. Pertsev , V. G. Koukhar , Phys. Rev. B ‐ Condens. Matter Mater. Phys. 2001, 64, 214103.

[19] C. Wang , J. Jiang , X. Chai , J. Lian , X. Hu , A. Q. Jiang , ACS Appl. Mater. Interfaces 2020, 12, 44998. 32914949 10.1021/acsami.0c13534

[20] L. Li , L. Xie , X. Pan , Rep. Prog. Phys. 2019, 82, 126502. 31185460 10.1088/1361-6633/ab28de

[21] C. T. Nelson , P. Gao , J. R. Jokisaari , C. Heikes , C. Adamo , A. Melville , S.‐H. Baek , C. M. Folkman , B. Winchester , Y. Gu , Y. Liukui Zhangenge , W. Lilong‐Qing , C.‐B. Eomdarrell , G. Schlomand , X. Pan , Science 2011, 334, 968. 22096196 10.1126/science.1206980

[22] T. Schenk , E. Yurchuk , S. Mueller , U. Schroeder , S. Starschich , U. Böttger , T. Mikolajick , Appl. Phys. Rev. 2014, 1, 041103.

[23] D. Damjanovic , Rep. Prog. Phys. 1998, 61, 1267.

[24] T. M. Raeder , K. Bakken , J. Glaum , M. A. Einarsrud , T. Grande , AIP Adv. 2018, 8, 105228.

[25] F. Xu , S. Trolier‐McKinstry , W. Ren , B. Xu , Z.‐L. Xie , K. Hemker , J. Appl. Phys. 2001, 89, 1336.

[26] X. Ren , Nat. Mater. 2004, 3, 91. 14716304 10.1038/nmat1051

[27] M. Kohli , P. Muralt , N. Setter , Appl. Phys. Lett. 1998, 72, 3217.

[28] I.‐E. Nylund , T. M. Raeder , P. E. Vullum , T. Grande , J. Appl. Phys. 2021, 129, 095304.

[29] H. Stadler , P. Zachmanidis , J. Appl. Phys. 1963, 34, 3255.

[30] S. Jesse , P. Maksymovych , S. V. Kalinin , Appl. Phys. Lett. 2008, 93, 11.

[31] N. Borodinov , SHO fitter combining NN and LS optimizer, https://github.com/nickborodinov/sho_fitter (accessed: March 2020).

[32] K. He , X. Zhang , S. Ren , J. Sun , in 2016 IEEE Conference on Computer Vision and Pattern Recognition (CVPR), IEEE, Piscataway, NJ 2016, pp. 770–778.

[33] T. M. Reader , U. Hanke , E. Halvorsen , T. Grande , Smart Mater. Struct. 2020, 29, 115039.

[34] J. F. Scott , A. Hershkovitz , Y. Ivry , H. Lu , A. Gruverman , J. M. Gregg , Appl. Phys. Rev. 2017, 4, 4.

[35] L. Landau , E. Lifshitz , Phys. Z. Sowjetunion 1935, 8, 101.

[36] C. Kittel , Rev. Mod. Phys. 1949, 21, 541.

