# Supplementary material for: High Velocity, Low‐Voltage Collective In‐Plane Switching in (100) BaTiO3 Thin Films
Source: Adv Sci (Weinh). 2022 Aug 28;9(29):2201530. doi: 10.1002/advs.202201530 (PMC9561770; doi:10.1002/advs.202201530)
Supplement: Supplementary file 1 — Supporting Information [file ADVS-9-2201530-s001.pdf]

ADVANCED SCIENCE
Open Access

Supporting Information

for *Adv. Sci.*, DOI 10.1002/advs.202201530

High Velocity, Low-Voltage Collective In-Plane Switching in (100) $BaTiO_3$ Thin Films

Trygve M. Ræder, Shuyu Qin, Michael J. Zachman, Rama K. Vasudevan, Tor Grande and Joshua C. Agar**

Supporting information:
High Velocity, Low-Voltage Collective In-Plane Switching in (100) $BaTiO_3$ Thin Films
Trygve M. Ræder, Shuyu Qin, Michael J. Zachman, Rama K. Vasudevan, Joshua C. Agar, and Tor Grande

S1: Kelvin-probe force microscopy

For KPFM the potential was adjusted on both electrodes simultaneously, one electrode with a positive bias, the other with a negative. The potential difference was adjusted in a stepwise fashion as an KPFM image was collected, collecting 16 line-scans at each bias. A DC-dependent offset in the KPFM data was observed. This is likely due to crosstalk with the DC bias, and a linear correction has been applied.

KPFM line-scans between the electrodes at different bias potentials are shown in Figure S1. The line-scans show an uneven distribution of the potential across the sample, particularly when a large bias is applied. For a positive bias, there is a particular accumulation of electric field at the left electrode, while for a negative bias a similar concentration is observed by the right electrode. This is similar to results from ceria films with coplanar electrodes, where water splitting on the surface was observed[2, 1]. The normalized piezoresponse across the sample is also shown. The piezorespnse reflects the distribution of the AC field driving the piezoresponse through the film compared to the surface potential potential measured by KPFM. The average piezoresponze is comparatively more homogeneous across the sample.

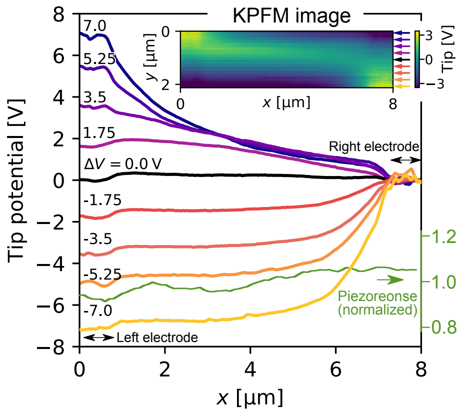

Figure S1: Potential profiles measured across the sample at different applied bias. The individual curves show line-scans from the KPFM image shown in the inset. The individual line-scans are labelled according to the potential difference between applied between the electrodes (ΔV). The line-scans have been offset to show the relative potential with respect to the right electrode. The normalized average PFM intensity across the film is shown for comparison.

The KPFM scans confirm that the potential between the electrodes reflects the applied potential. An accumulation of potential by the electrodes is expected based on an electrostatic model [3], but the distribution is expected to be symmetric at the two electrodes, and smaller than what is observed here.

Furthermore, the distribution of the electric field across the samples is expected to reflect the amplitude in the BEPFM, as a larger electric field will give a larger piezoresponse. However, the amplitude observed in the BEPFM is much more homogeneous across the sample, indicating a more linear distribution of the electric field. The results from KPFM is likely affected by water splitting on the surface and a distribution of related species [5, 4] causing a build-up of field by the positive electrode [2, 1]. Evaluating the piezoresponse amplitude across the sample therefore offers an alternative approach to estimating the electric field distribution in the film, and confirms an even distribution. Future applications should consider encapsulation in order to avoid electrochemical reactions at the surface.

References

[1] J. Ding, E. Strelcov, S. V. Kalinin, and N. Bassiri-Gharb. Electrochemical reactivity and proton transport mechanisms in nanostructured ceria. *Nanotechnology*, 27(34), 2016.

[2] Jilai Ding, Evgheni Strelcov, Sergei V. Kalinin, and Nazanin Bassiri-Gharb. Spatially Resolved Probing of Electrochemical Reactions via Energy Discovery Platforms. *Nano Letters*, 15(6):3669–3676, 2015.

[3] Trygve M Reader, Ulrik Hanke, Einar Halvorsen, and Tor Grande. A unified approach for the calculation of in-plane dielectric constant of films with interdigitated electrodes. *Smart Materials and Structures*, 29(11):115039, 2020.

[4] Evgheni Strelcov, Anton V. Ievlev, Stephen Jesse, Ivan I. Kravchenko, Vladimir Y. Shur, and Sergei V. Kalinin. Direct probing of charge injection and polarization-controlled ionic mobility on ferroelectric LiNbo3 surfaces. *Advanced Materials*, 26(6):958–963, 2014.

[5] Evgheni Strelcov, Stephen Jesse, Yen Lin Huang, Yung Chun Teng, Ivan I. Kravchenko, Ying Hao Chu, and Sergei V. Kalinin. Space- and time-resolved mapping of ionic dynamic and electroresistive phenomena in lateral devices. *ACS Nano*, 7(8):6806–6815, 2013.

S2: All BE-PFM frames

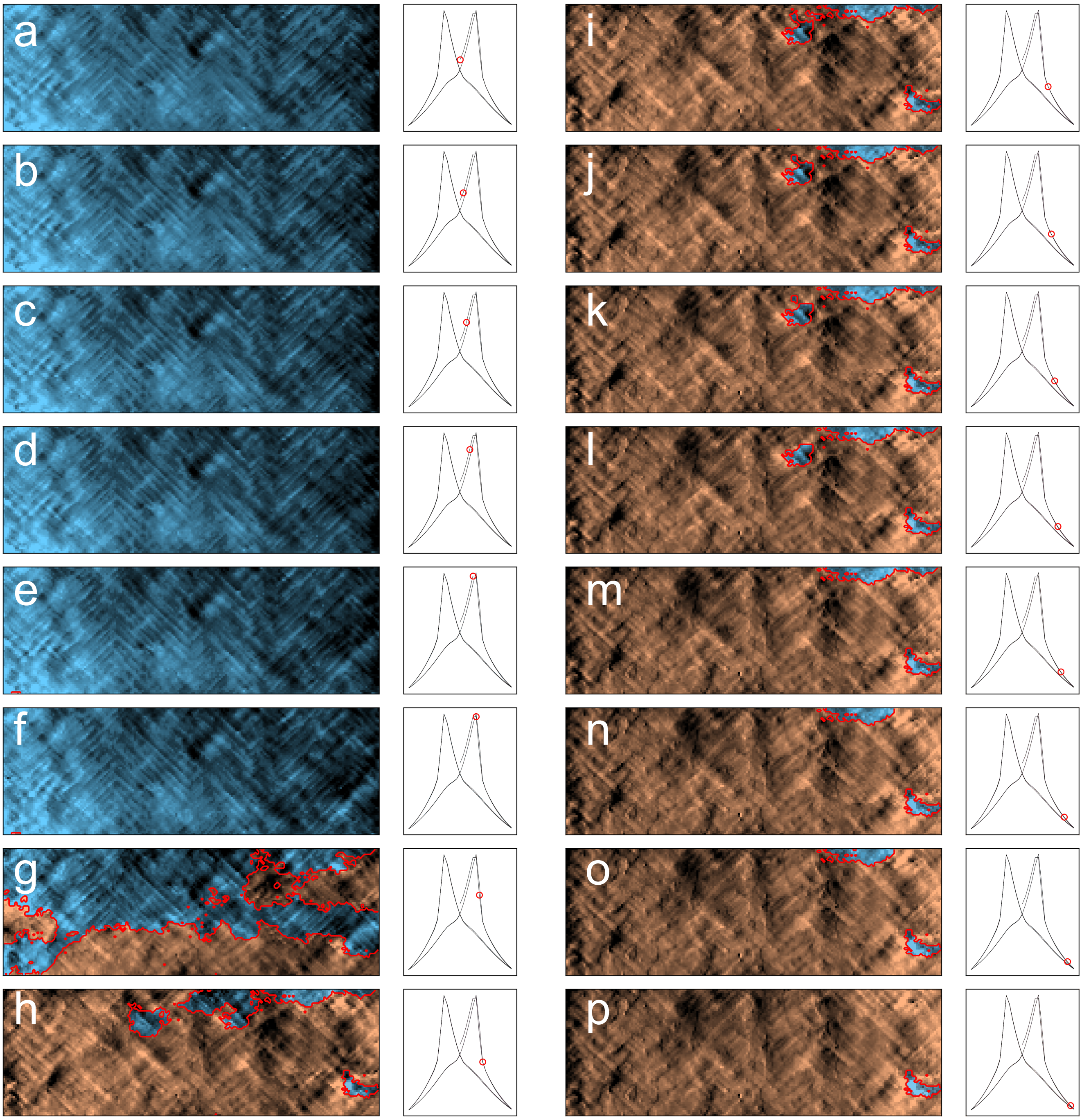

Figure S2: First 16 BE-PFM frames.

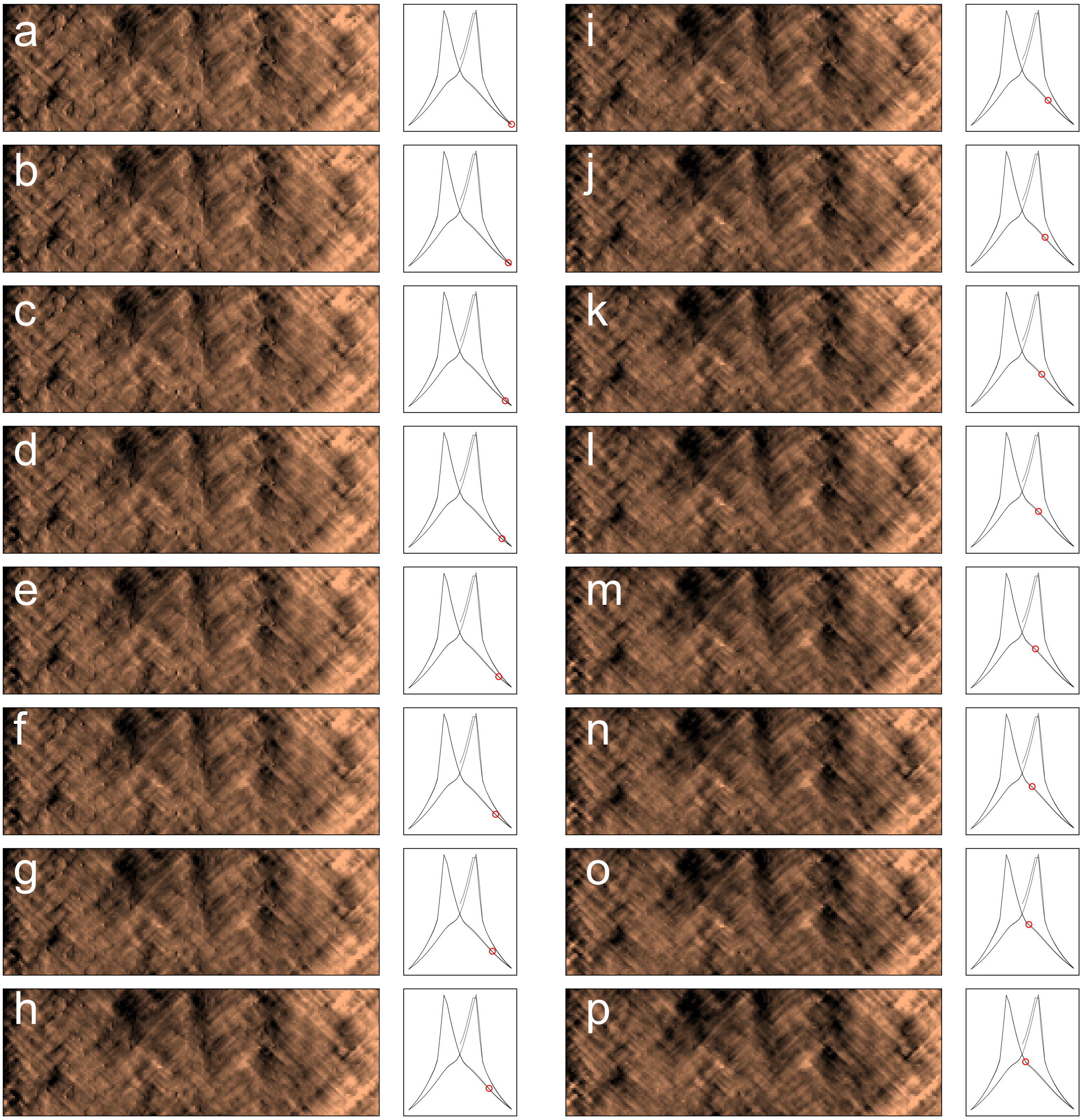

Figure S3: BE-PFM frames 17-32.

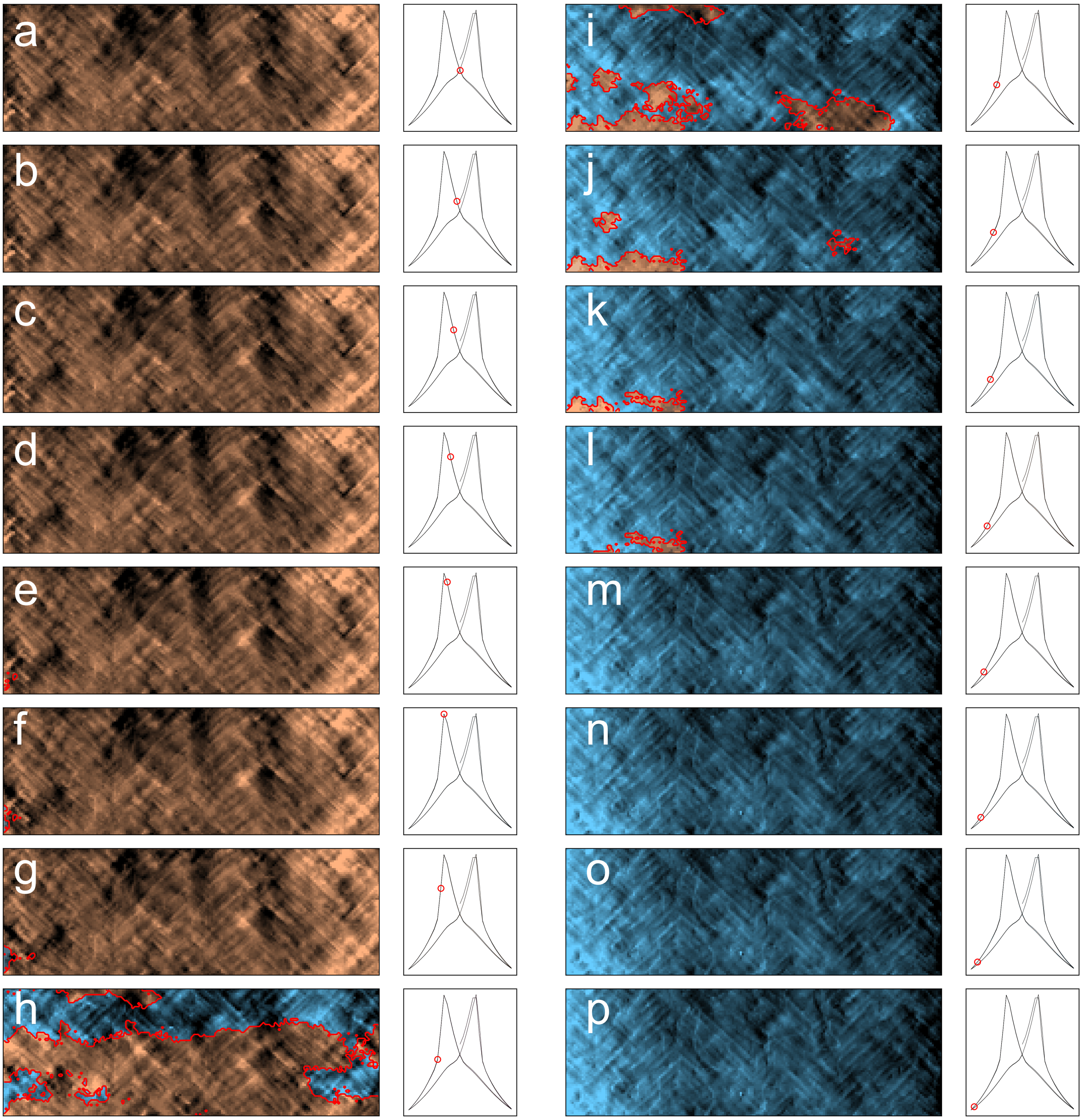

Figure S4: BE-PFM frames 33-48.

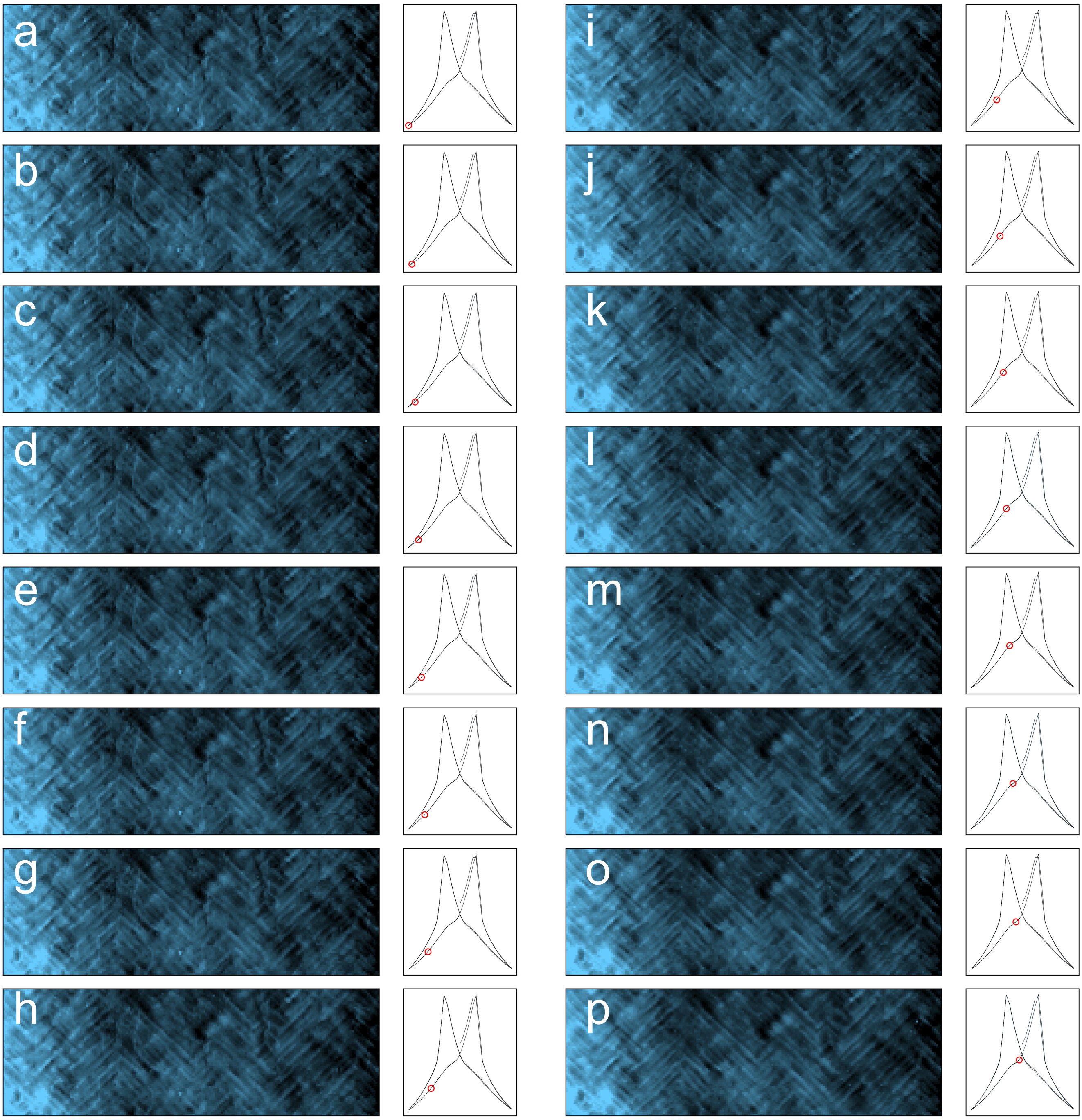

Figure S5: BE-PFM frames 49-64.

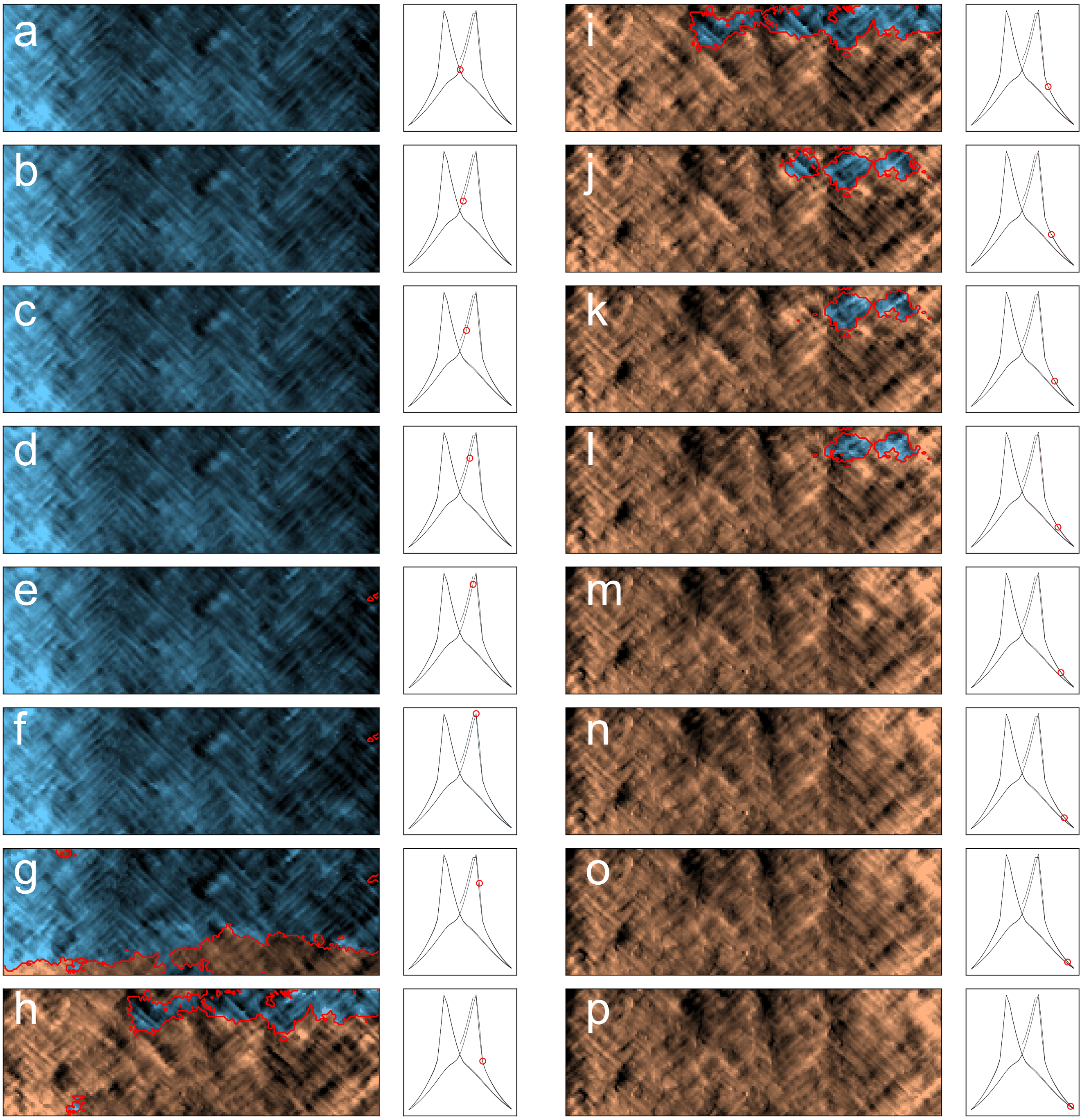

Figure S6: BE-PFM frames 65-80, also included in the main report.

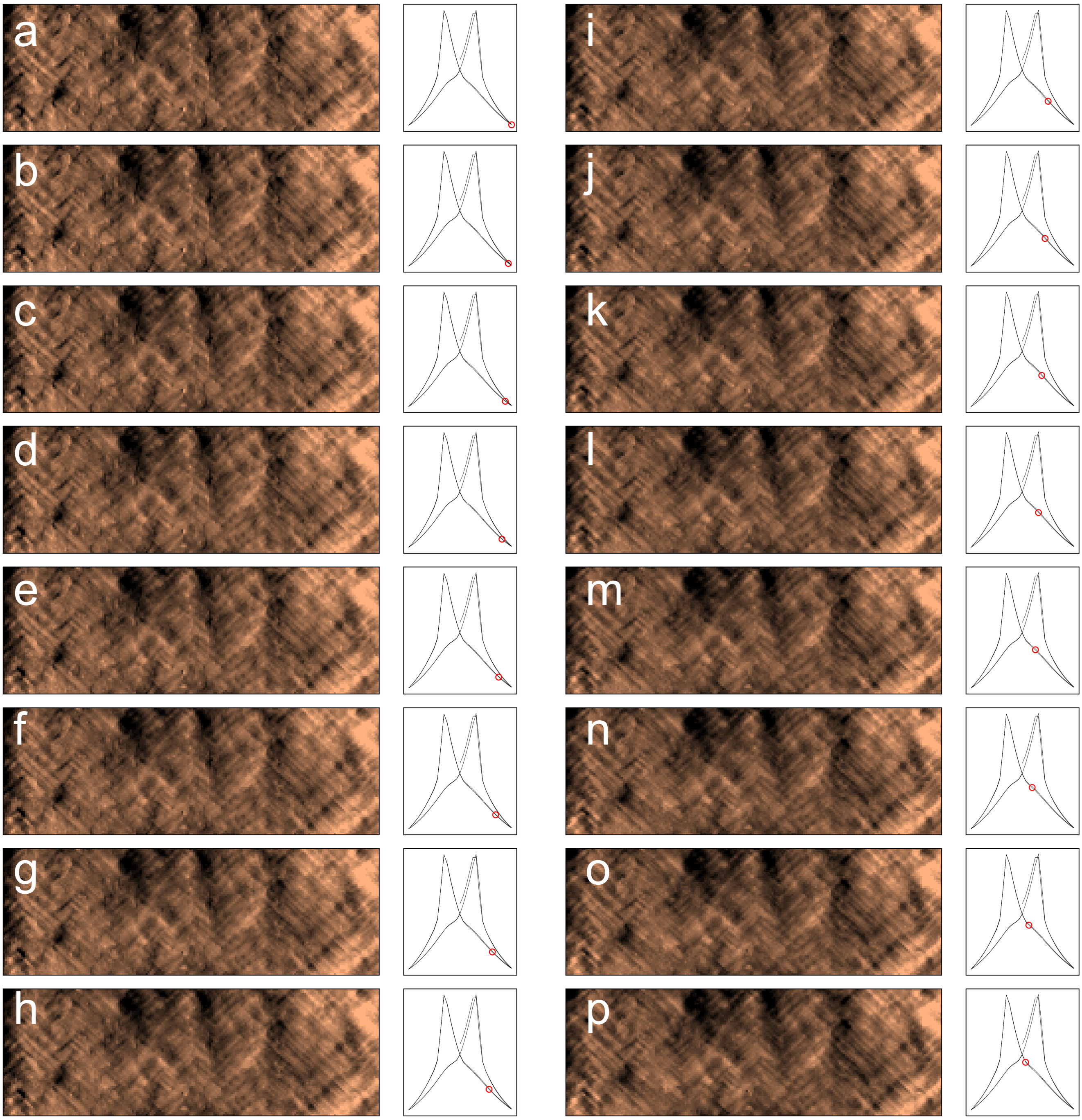

Figure S7: BE-PFM frames 81-96.

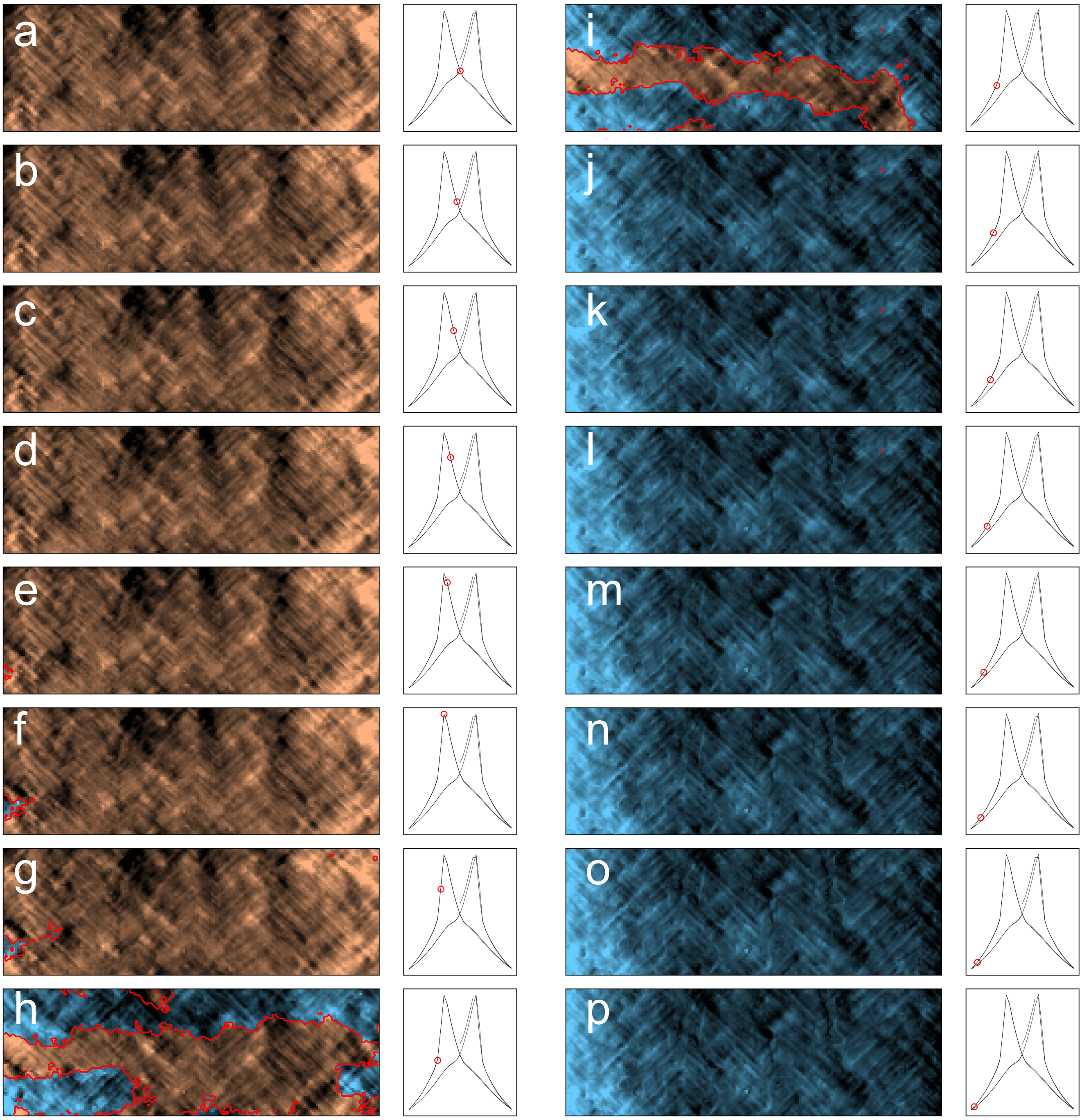

Figure S8: BE-PFM frames 97-112.

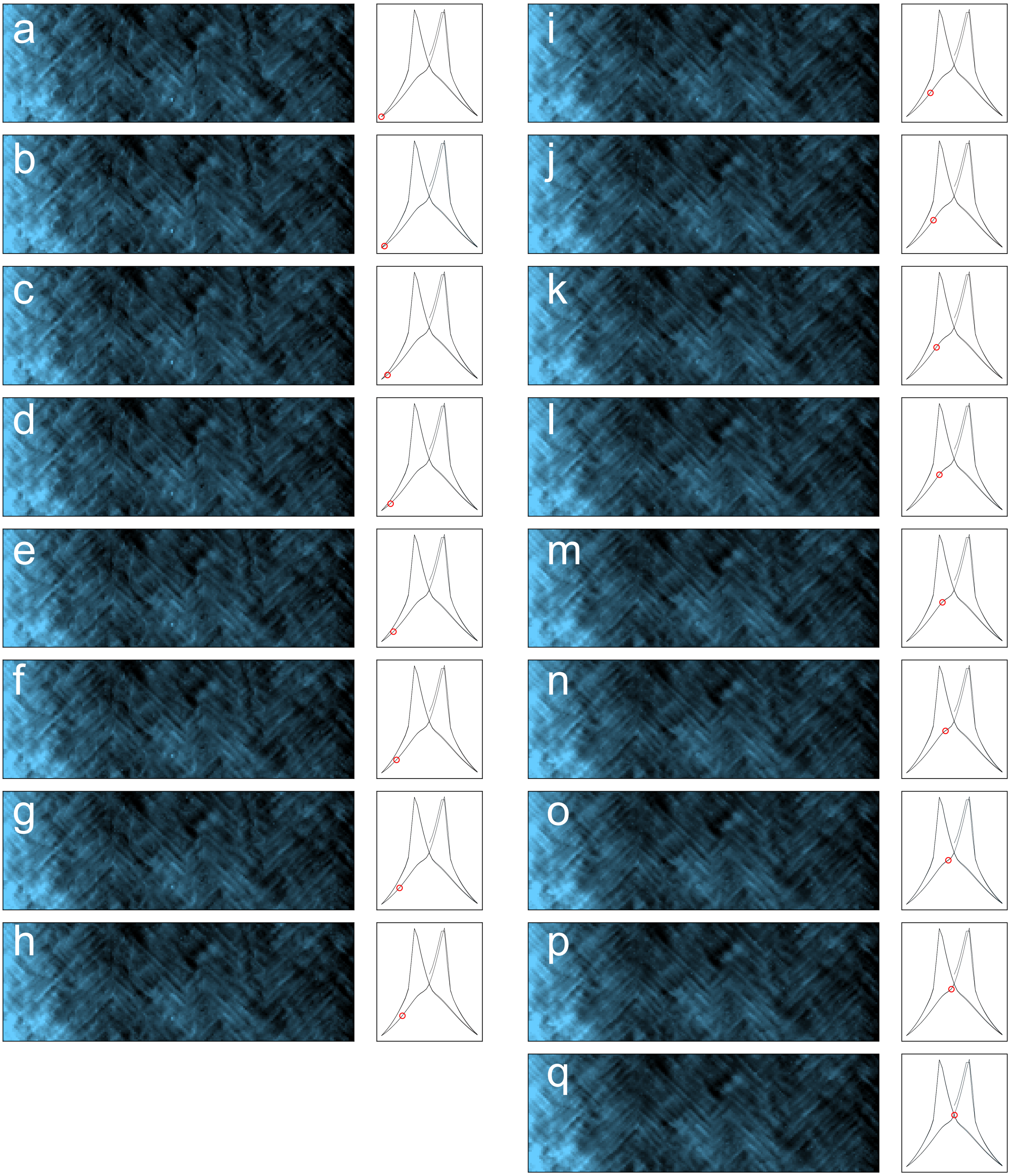

Figure S9: BE-PFM frames 113-129.

S3: Changes after complete cycles

The majority of the domain structure remains after switching twice (back to the original state). This is shown in Figure S10, where the small changes that do occur are highlighted.

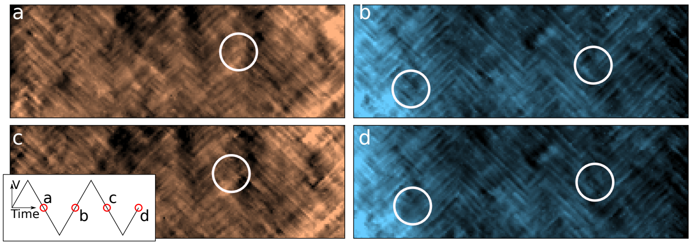

Figure S10: The domain pattern after the **(a)** first, **(b)** second, **(c)** third and **(d)** fourth switching event, as shown in the inset. All images are at zero bias. The majority of the domain pattern is preserved after an even number of switching events. The circles indicate regions where this is not the case.
